# Living under siege: resilience, hopelessness, and psychological distress among Palestinian students in the Gaza Strip

**DOI:** 10.1017/gmh.2021.37

**Published:** 2021-10-12

**Authors:** Guido Veronese, Alessandro Pepe, Marwan Diab, Yasser Abu Jamey, Ashraf Kagee

**Affiliations:** 1 University of Milano-Bicocca, Milan, Italy; 2 Stellenbosch University, Stellenbosch, South Africa; 3 Gaza Community Mental Health Program, Gaza, Palestine

**Keywords:** Hopelessness, mental health, Palestinian students, resilience, siege

## Abstract

**Background:**

Moving from an approach oriented to adaptation and functioning, the current paper explored the network of cumulative associations between the effects of the siege and resilience on mental health.

**Methods:**

We sought to explore the impact of the siege on psychological distress (anxiety, depression, and stress) and the moderating effect of resilience and hopelessness in a sample of 550 Palestinian university students. We hypothesized that the siege effect would impact psychological distress so that the more people were affected by the siege, the more mental symptoms of common mental disorders they would report. We also expected that the siege would negatively impact both resilience and participants' hopelessness.

**Results:**

Findings showed that higher scores on the scale measuring effect of the siege were associated with hopelessness. Furthermore, living under siege compromised participants’ resilience. The more the siege affected individuals, the lower resilience were protecting participants mental health and the more hopelessness was exposing them to anxiety, stress, and depression.

**Conclusion:**

Our findings draw attention to how the ongoing violation of human rights influences people's mental health in Gaza. Implications for clinicians and policymakers are discussed.

The Gaza Strip, an area of the occupied Palestinian territories, has endured 13 years of almost complete siege by the Israeli army (Handley and Ismail, 2010). During this period, Gaza has endured three major Israeli attacks in 2008, 2012, and 2014, resulting in thousands of deaths, injuries, and disability. Israeli aggression has created a chronic humanitarian crisis characterized by food insecurity, limited access to health care, lack of electricity and fuel, unemployment, and widespread poverty, as well as an environmental emergency due to air, land, and water pollution (Winter, 2016). The area is completely closed and isolated by walls and fences separating it from Israel in the northern and eastern regions and from Egypt in the south, by sea due to an Israeli-imposed limitation on Palestinian vessels sailing more than two nautical miles from the western coasts, and by airspace as drones constantly fly over the area and regularly destroy strategic sites and civilian dwellings that are considered a threat for the Israeli security (Smith, 2015, 2016).

As for the definition used by the United Nations to define siege, blockade is ‘an act of war whereby one party blocks entry to or departure from a defined part of an enemy's territory, most often its coasts’ (https://www.britannica.com/topic/blockade-warfare). Blockades are regulated by international law and custom and require advance warning to neutral states and impartial application. Yemen and Gaza are the most well-known and recognized cases in the contemporary warfare (Erakat, 2011; Fink, 2017).

Office of the High Commissioner for Human Rights special rapporteur Idriss Jazairy recently stated in a briefing at the UN Human Rights Council that civilians affected by blockades cannot benefit from the protection of the Geneva Convention, which is aimed at safeguarding victims in war time (UN News, 2018). Consequently, civilian victims of blockade and chronic war are likely to develop psychological and physical burdens that can undermine individuals' and social functioning (Thabet *et al*., 2008; Hijazi *et al*., 2019).

Joma'a and Thabet (2015) have identified a number of stressors that may affect students in Gaza living under siege and chronic warfare. Of the 367 participants in their study, 92% stated that they were affected by sharp price increases due to the siege; 333 reported to have seen their studies compromised due to cut-off of electricity and shortage of gas (83.5%); and 285 students reported that their families were unable to help in paying fees for the university because of lack of money (71.4%). Finally, 58% of the respondents was found not to be able to receive proper medical care.

Thabet and his colleagues reported several psychological sequelae as a consequence of the blockade over the years. In fact, stressors resulted in depressive symptoms and anxiety in more than 10% of the interviewed men and women in Gaza (Joma'a and Thabet, 2015). Women reported somatic, obsessive compulsive, and phobic symptoms more than men, while people who scored higher on the items related to the impact of the siege also reported more posttraumatic symptoms (66.6% of the sample), depression (42.3% moderate, 3.1% severe), and psychosis (12%) (Thabet and Vostanis, 2005; Thabet *et al*., 2008; Lubbad and Thabet, 2009; Thabet *et al*., 2013). Accordingly, quality of life of Palestinians on the physical, psychological, and environmental domains was found to be among the lowest of any population in the world (Skevington *et al*., 2004; Mataria *et al*., 2009). In fact, the mean scores from the World Health Organization (WHO) index of quality of life ranged from about 68 points on a 100-point scale for the environment domain to a score slightly above 80 points for the physical domain (Hammoudeh *et al*., 2013). The mean quality of life scores for both samples were lower than the international mean, particularly in the environmental domain. Risk of suicide among university students living in Gaza was discriminated by the lack of social support, psychological distresses such as depression anxiety and chronic stress, and hopelessness. Boys were identified as more at risk to get suicided compared to girls (Veronese *et al*., 2021).

Furthermore, in a study by Thabet and colleagues, participants from Gaza villages and refugee camps were found more affected by the siege than individuals from urban areas mainly because of economic restraint and lack of material resources. On the contrary, participants who showed greater spiritual well-being and social support were found as more resilient (see below this paragraph) (Thabet *et al*., 2009). Among a sample of 600 students in Gaza, 60% rated their quality of life as ‘poor’ or ‘very poor’ and attributed this to the siege (Ellessi *et al*., 2019). They reported sadness and hopelessness due to a sense that an end to the siege was not in sight and that their lives would not progress as a consequence of the situation (Elessi *et al*., 2019).

Yet, among 1068 households in the Gaza Strip, resilience scores were positively correlated with positive emotions comprising enthusiasm, alertness, determination, and pride. Higher levels of education and socio-economic status were associated with increased resilience among study participants (Kteily-Hawa *et al*., 2020). In addition, a group of 400 Palestinian adolescents was found to use coping strategies such as developing self-reliance (58.9%), accessing social support (45.5%), and family support (40.2%) to adjust to traumatic experiences (Thabet *et al*., 2014).

To our knowledge, no studies have provided a conceptual understanding of the effects of the siege on resilience and hope, resulting in civilians' mental well-being. The current study sought to test the above described conceptual model in a population affected by long-term blockade and warfare.

Using a socio-ecological perspective of functioning (von Lindern *et al*., 2017), the current paper explored the network of cumulative associations between the effects of the siege (i.e. deprivation of economic aspect, social dimension, and loss of basic rights) and resilience. Furthermore, we sought to explore the impact of siege and resilience on hopelessness and psychological distress (anxiety, depression, and stress) in a sample of Palestinian participants. We hypothesized that the personal experiences of siege would impact various psychological dimensions so that the more people were affected by the siege the more mental symptoms of common mental disorders they would report. We also expected that the circumstances of siege would impact both resilience and participants' sense of hopelessness.

## Method

### Sample and procedure

A convenience sample of 550 Palestinian university students was recruited from three different main universities in Gaza, namely, Al Azar, Al Aqsa, and Islamic University. Females comprised of 55.1% of the participants and mean age was 20.5 ± 1.76 (min–max 18–29). The participants in the sample were mainly from cities (75.1%, *n* = 393) and 18.7% (*n* = 93) lived in rural areas. The majority (84.1%) was unmarried. The most common areas of study were education (42.0%) and nursing (8.2%). The inclusion criteria encompassed being Palestinian living in Gaza Strip and not to have traveled abroad prior to the study, being enrolled in a university or academic college during the research period.

Questionnaires were administered on-site by trained research assistants. The Palestinian Health Research Council granted the permission to approach the students through the Dean of Student Affairs and the Student Council facilitation at each university. Data collection took place during the students' free time from lecture to lecture. Faculty members at the various schools in each university presented the study to the students, while the researchers invited them to participate in the research. Groups of interested students were convened in halls at their respective universities and fully briefed about the study. Participants then completed the questionnaires throughout a pencil and paper methodology. Prior to participating in the study, all participants were informed about the research aims and provided written informed consent. Data were collected between 2018 and 2019. Four hundred thirty one out of 550 students were invited for the task, 431 agreed to take part and 114 declined or did not submit completed questionnaires, with a response rate of approximately 80% (Table 1).

**Table 1:** Summary of demographic data (*N* = 550)

Age	20.5 ± 1.76 (min–max 18–29)
Gender	55.1% female; 44.9% male
Location	75.1% cities; 18.7% rural areas; 6.2% other
Marital status	84.1% unmarried; 15.9% married

The research was conducted following the American Psychological Association's ethical guidelines (2010) and code of conduct, and the research protocol was approved by the Ministry of Health Ethic Committee in Gaza (Helsinki Ethic Committee Protocol no. PHRC/HC/284/17).

### Measures

*Resilience Scale* (RS; Wagnild and Young, 1993): The RS is a self-reported measure of interpersonal and intrapersonal protective resources that facilitate adaptation to adverse life events. In terms of the RS, resilience is described as an individual strength that helps people to adapt despite adversity (Wagnild, 2009). The measure consists of 25 items that measure five latent components of resilience, namely, equanimity, perseverance, self-reliance, purpose, and existential aloneness. In the current study, the internal reliability of scores (*α*; Cronbach, 1951) were as follows: equanimity [*α* = 0.62 (95% confidence interval (CI) 0.558–0.679)], perseverance [*α* = 0.81 (95% CI 0.791–0.835)], self-reliance [*α* = 0.67 (95% CI 0.617–0.707)], meaningfulness [*α* = 0.61 (95% CI 0.555–0.652)], and existential aloneness [*α* = 0.74 (95% CI 0.706–0.764)].

*Beck Hopelessness Scale* (BHS; Beck and Weissman, 1974): The BHS is a quantitative inventory developed to measure hopelessness in adults between 17 and 80 years. The model of measurement consists of 20 items and three latent components: lack of expectations, negative feelings about the future, and loss of motivation. All items are scored on a true–false response scale. Cutoff scores classification to BHS cumulate score were: minimal (<3); mild (4–8), moderate (9–14), and severe (15–20). Although past studies indicate that the BHS is moderately correlated with depression (Beedie, and Kennedy, 2002; Courtney *et al*., 2008; Toussaint *et al*., 2008), evidence exists that hopelessness is more strongly related to suicidality than to depression (Kovacs, and Garrison, 1985). The WHO has suggested that hopelessness is a risk factor for suicidal intention (WHO, 2014; Hagan *et al*., 2015) and a crucial aspect in the context of life satisfaction, compliance, and emergency settings (Shek, and Li, 2016). In the current study, the internal reliability of scores (*α*; Cronbach, 1951) are follows: expectation [*α* = 0.73 (95% CI 0.688–0.751)], feelings about the future [*α* = 0.76 (95% CI 0.728–0.785)], and loss of motivation [*α* = 0.65 (95% CI 0.596–0.682)].

*Depression Anxiety Stress Scale* (DASS-21; Antony *et al*., 1998): The DASS-21 is a clinical measure originally developed with the aim of providing an evaluation of the core symptoms of depression and anxiety in a non-clinical sample (Lovibond and Lovibond, 1995a). The content of the DASS was identified *a priori* on the basis of clinical consensus and was then empirically refined using factor analysis. The questionnaire consists of a pool of 21 items aimed at measuring three different symptomatologic areas of mental health, namely, (a) depression, low self-esteem, and dysphoria; (b) somatic and subjective symptoms of anxiety, as well as responses of fear; and (c) stress, evaluating irritability, impatience, tension, and persistent arousal (Lovibond and Lovibond, 1995b). Cut-off scores of DASS-21 can be used as an assessment of disturbance (i.e. individuals who may need a specific diagnosis) as it is able to identify individuals experiencing symptoms and at high risk of further problems (Weiss *et al*., 2015). In this study, the internal consistencies of different factors of the DASS-21 (*α*; Cronbach, 1951) were *anxiety* [*α* = 0.82 (95% CI 0.796–0.840)], *depression* [*α* = 0.81 (95% CI 0.790–0.834)], and *stress* [*α* = 0.77 (95% CI 0.743–0.798)].

*Gaza Siege Checklist* (GTCL; Thabet *et al*., 2008): The GCMHP is a self-reported checklist that consists of 21 items covering a range of common situation that might be affected by living under siege. The items were developed to map different domains of daily life such as family aspects, social aspect, education, and economic issues. The total score can be used as a cumulate measure of the impact of the siege on the life of the participants. It ranges from 0 to 21 with higher score indicating a higher impact of the siege. Some examples of the items are: ‘I feel like I'm living in a big prison’, ‘I stopped buying my needs’, and ‘I quit my social visits’. In this study, the internal consistency of the GCMHP (*α*; Cronbach, 1951) was *α* = 0.78 (95% CI 0.749–0.801).

### Modeling and strategy of data analysis

**Fig. 1.**
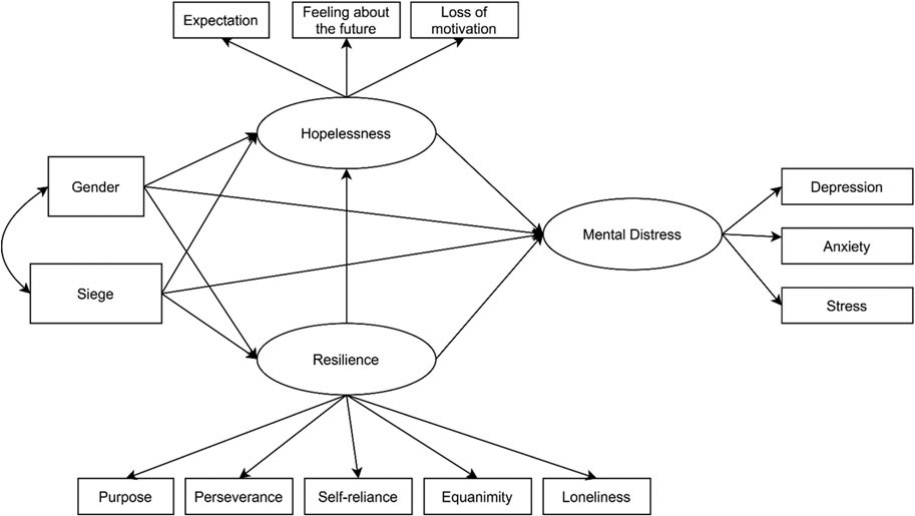
Conceptual model of resilience, hopelessness, and mental health.

To evaluate the conceptual model of hopelessness and resilience in relation to mental health, we used structural equation modeling (SEM) (Guo *et al*., 2009; Collier, 2020) to estimate the magnitude and direction of structural associations among the variables. SEM is a type of path analysis that is known to perform well in terms of the validity and reliability of the estimated effects (Hair *et al*., 2017). It represents a confirmatory rather than an exploratory approach that integrates measurement with a conceptual perspective by allowing both empirical indicators and latent factors to be estimated. In the current study, the conceptual model (see Fig. 1) was composed of three latent endogenous variables, two exogenous dimensions, and 11 empirical indicators (see Fig. 1).

Moving from left to right, the two exogenous constructs were the impact of the siege as measured by the cumulate score of the GTCL and the gender of the participants. With regard to endogenous latent variables, hopelessness was operationalized by means of loss of motivation, expectation, and feeling about the future. Resilience consisted of equanimity, perseverance, self-reliance, purpose, and existential loneliness. Finally, the last endogenous latent variable was participants' mental health assessed through depression, anxiety, and stress as the empirical indicators. To better evaluate the cumulative network of association among variables, estimated total effects were broken down into direct and indirect effects (Mitchell, 1992).

To test the robustness of the conceptual model, we evaluated the following goodness-of-fit indices: root mean square error of approximation (RMSEA < 0.08; Hu and Bentler, 1999); normed fit index (NFI > 0.95) (Marsh *et al*., 2013), Tucker–Lewis index (TLI > 0.95) (Morin *et al*., 2013), and comparative fit index (CFI > 0.95) (Morin *et al*., 2013). In keeping with best practices in SEM (e.g. Thakkar, 2020), we estimated confidence limits using both Monte Carlo simulation and bootstrapping methods with a set of random samples (*k* = 500). We calculated given indirect effects for each of the *k* samples and the mean value for the selected pool of samples. Please note that CIs referred to unstandardized estimates, on the contrary in the ‘Result’ section standardized weights will be reported and then discussed. We also computed Mahalanobis' distance (*p* < 0.001) to identify multivariate outliers, finding that no cases needed to be removed from the dataset. Finally, we assessed the data for normality of distribution. None of the score sets displayed kurtosis or skewness values exceeding the recommended limits [−1, +1], and consequently the maximum likelihood method (Gath and Hayes, 2006) was adopted to estimate the parameters for the SEM analysis. Software used for all analyses was Amos 23.0 (Arbuckle, 2014).

## Results

### Siege, hopelessness, and mental health: general overview and descriptive statistics

**Fig. 2.**
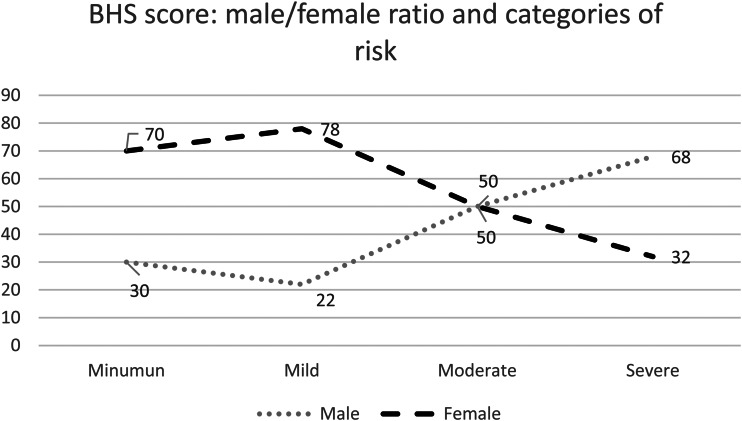
Risk of suicide rates according to hopelessness scale cut-off scores. Data were expressed in %. Within each category of risk, percentage of male and female were reported. BHS = Beck Hopelessness Scale. Minimal (*n* = 30, 5.5%), mild (*n* = 180, 32.7%), moderate (*n* = 255, 46.4%), and severe (*n* = 85, 15.4%).

In terms of the first aim, descriptive results of GSCS, BHS, and DASS-21 were presented separately to describe the overall psychological condition of the participants. Aggregate cumulate scores were used in multivariate statistics to model the data. Concerning the effect of the prolonged condition of the siege, the items that were most commonly endorsed in the scorable direction were: ‘Social visits are less than before’ (74.3%), ‘My monthly income has decreased’ (85.2%), and ‘I feel I am in a big prison’ (78.6%). On the contrary, the least frequently endorsed items were: ‘I had suffering of not be able to receive proper medical care’(15.6%), ‘I went to organization to get food’ (19.4%), and ‘I started doing the paper for immigration’ (15.4%). With regard to the hopelessness score, the overall data suggest a general tendency to high scores on the BHS: minimum (*n* = 30, 5.5%), mild (*n* = 180, 32.7%), moderate (*n* = 255, 46.4%), and severe (*n* = 85, 15.4%). According to the cut-off points identified by Beck (1974) and colleagues that linked hopelessness to suicidal ideations shown in Fig. 2, we report the percentages of male and female participants belonging to each risk group ranging from minimal to severe risk of suicide (McMillan *et al*., 2007; Granö *et al*., 2017). For instance, in the group at minimum risk (*n* = 30), 70% were female and 30% were male. On the contrary, in the group at severe risk (*n* = 85), 68% were male and 32% female. In the moderate and severe categories, there are much higher rates of males (up to 60%) whereas in minimum and mild categories the percentage of females increased up to approximately 80%. As the level of risk of suicidal ideations increased, the percentage of male was higher.

Finally, the results of DASS-21 categorized according to cut-off points are summarized in Table 2.

**Table 2:** Summary symptoms severity according to cut-off points of DASS-21 (*N* = 550)

	Depression (%)	Anxiety (%)	Stress (%)
Normal risk	23.1	14.5	35.8
Mild risk	13.1	9.3	17.8
Moderate risk	40.5	14.9	25.8
Severe risk	8.9	18.5	15.1
Extreme risk	14.4	42.7	5.5

The main descriptive statistics along with zero-order correlations among scores on hopelessness, resilience, and mental health are presented in Table 3, followed by the final structural equation model.

**Table 3:** Main descriptive statistics and zero-order correlation among siege, hopelessness, resilience, and mental health scores (*N* = 550)

	1	2	3	4	5	6	7	8	9	10	11	12	13
1. Siege scale	−												
2. Expectation	0.265**	−											
3. Feeling about the future	0.251**	0.290**	−										
4. Loss of motivation	0.369**	0.449**	0.434	−									
5. Equanimity	−0.271**	−0.234**	−0.223**	−0.524**	−								
6. Perseverance	−0.133**	−0.056	−0.047	−0.242**	0.531**	−							
7. Self-reliance	−0.249**	−0.152**	−0.137**	−0.431**	0.709**	0.556**	−						
8. Purpose	−0.224**	−0.091*	−0.076	−0.322**	0.628**	0.587**	0.618**	−					
9. Existential loneliness	−0.215**	−0.116**	−0.102*	−0.425**	0.687**	0.662**	0.725**	0.690**	−				
10. Anxiety	0.360**	0.213**	0.205**	0.412**	−0.238**	−0.120**	−0.198**	−0.176**	−0.264**	−			
11. Depression	0.373**	0.290**	0.284**	0.531**	−0.337**	−0.173**	−0.250**	−0.208**	−0.309	0.757**	−		
12. Stress	0.337**	0.226**	0.217**	0.393**	−0.181**	−0.077	−0.066	−0.118**	−0.177**	0.778**	0.776**	−	
13. Gender	−0.292**	−0.119**	−0.104*	−0.250**	0.225**	0.105*	0.285**	0.182**	0.168**	−0.008	−0.079	0.015	−
Mean	10.60	3.11	3.11	3.21	8.54	9.74	17.35	12.55	18.19	9.03	8.32	9.27	−
Standard deviation	3.98	1.22	1.23	2.09	2.50	2.59	4.56	3.29	4.51	4.90	4.61	4.26	−
Skewness	−0.11	0.12	0.10	0.57	−0.57	−0.48	−0.98	−0.59	−0.92	0.36	0.33	0.28	−

*p < 0.05, **p < 0.01, baseline male = 1.

The results of the correlational analysis indicate that the effects of the siege were positively associated with both hopelessness score (with *r* value ranging from 0.25 to 0.37) and mental distress (with *r* value ranging from 0.34 to 0.37). Also, negative small to medium statistically significant correlations were found between the effects of siege and measures of resilience. With regard to resilience, the analysis reveals a negative and statistically significant association with both the hopelessness scale and mental distress (i.e. depression, anxiety, and stress), meaning that the higher the resilience score, the better the general mental health condition of participants were. Finally, the gender of the participants was found to be associated with almost all variables, with females reporting more resilience and less hopelessness compared to males.

### Modeling the relationship among siege, hopelessness, resilience, and mental health

**Fig. 3.**
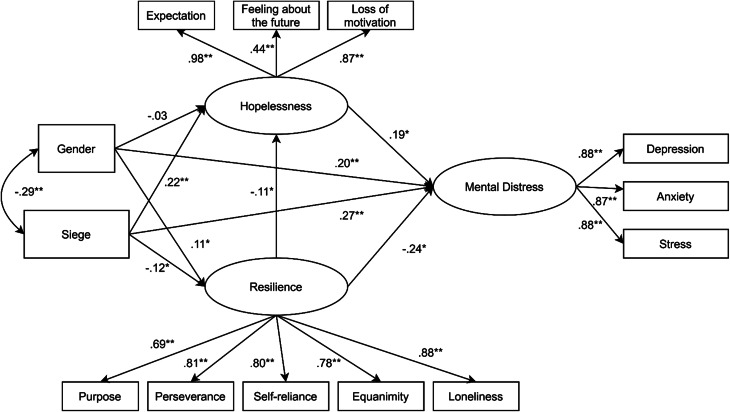
Results of structural equation model of siege, resilience, hopelessness, and mental health (*N* = 550), standardized direct effects were reported. *p < 0.05; **p < 0.01.

A more detailed description of the relationship among the variables is provided in Fig. 3.

In terms of the general fit between the conceptual model and the empirical data, the goodness of fit indices supported the acceptance of the model: χ^2^(52) = 257.2, *p* < 0.05, NFI = 0.955, NNFI = 0.964, CFI = 0.965, RMSEA = 0.075, 90% CI 0.065–0.085. Regarding the consequences of the siege, statistically significant positive total standardized effects were found in relation to hopelessness (*β* = 0.24, *p*  = 0.004, 90% CI 0.048–0.091) and mental distress (*β* = 0.34, *p* = 0.004, 90% CI 0.270–0.418). Interestingly, the consequence of the siege had a statistically significant negative total standardized effect also on resilience (*β* = −0.12, *p*  = 0.018, 90% CI −0.180 to −0.040) meaning that the outcomes of living under the siege affected all psychologically considered domains. In line with the traditional view of resilience as a protective factor, the results suggested statistically significant negative total effects with both hopelessness (*β* = −0.11, *p*  = 0.020, 90% CI −0.061 to −0.010) and mental health conditions (*β* = −0.24, *p*  = 0.013, 90% CI −0.324 to −0.161), meaning that higher resilience scores, and lower hopelessness scores were associated with better the mental status. Hopelessness was found to be associated with mental health with a statistically significant positive direct effect: *β* = 0.19, *p* = 0.005, 90% CI 0.374–0.900. Finally, gender was associated with the context of siege *β* = −0.29, *p*  = 0.003, 90% CI −2.88 to −1.82 with males reporting more mental health consequences of living in that condition. Similarly, total statistically significant positive effects were found in relation to resilience (*β* = 0.15, *p*  = 0.007, 90% CI 0.694–1.72). With regard to mental distress, a positive direct effect (*β* = 0.20, *p* = 0.003, 90% CI 1.02–2.16) was found, however the total standardized effect was not statistically significant (*β* = 0.06, *p*  = 0.120, 90% CI −0.35 to 0.983). Finally, gender resulted to be associated with hopelessness with a total direct statistically significant effect (*β* = −0.11, *p* = 0.003, 90% CI −0.441 to −0.117) was found between gender and hopelessness. Details about total, direct, and indirect effects are summarized in Table 4.

**Table 4:** Results of the structural equation model: summary of total, direct, and indirect effects

		Total effect			Direct effect			Indirect effect		
		*β*	*p*	90% CI [LB; UB]	*β*	*p*	90% CI [LB; UB]	*β*	*p*	90% CI [LB; UB]
Gender	Siege	−0.29	0.003	[−2.88; −1.82]	−0.29	0.003	[−2.88; −1.82]	−	−	−
	Hopelessness	−0.11	0.003	[−0.441; −0.117]	−0.03	0.463	[−0.247; 0.103]	−0.08	0.002	[−0.279; −0.136]
	Resilience	0.15	0.007	[0.694; 1.72]	0.11	0.012	[0.382; 1.51]	0.04	0.013	[0.093; 0.441]
	Mental distress	0.06	0.120	[−0.035; −0.983]	0.20	0.003	[1.02; 2.16]	−0.14	0.002	[−0.140; −0.816]
Siege	Hopelessness	0.24	0.004	[0.048; 0.091]	0.22	0.004	[0.044; 0.087]	0.02	0.025	[0.002; 0.008]
	Resilience	−0.12	0.018	[−0.180; −0.040]	−0.12	0.018	[−0.180; −0.040]	−	−	−
	Mental distress	0.34	0.004	[0.270; 0.418]	0.27	0.004	[0.198;0.346]	0.07	0.003	[0.047; 0.108]
Resilience	Hopelessness	−0.11	0.020	[−0.061; −0.010]	−0.11	0.020	[−0.061; −0.010]	−	−	−
	Mental distress	−0.24	0.004	[−0.325; −0.161]	−0.22	0.004	[−0.304; −0.142]	−0.02	0.015	[−0.042; −0.006]
Hopelessness	Mental distress	0.19	0.005	[0.374; 0.900]	0.19	0.005	[0.374; 0.900]	−	−	−

*β* = standardized effect; CI = confidence interval; LB = lower bound; UB = upper bound.

## Discussion

We tested the combined effect of the siege in Gaza on mental health resilience and hopelessness. Generally speaking, the novelty of our study emerged from the systematization of results in other studies that checked correlations between the variables we tested in our study: effects of siege, hopelessness and resilience, and mental distress. For the very first time, a study explored the associations between such pivotal variables and how the siege is contributing to mental distress via hopelessness and resilience.

Our findings demonstrated a crucial role of the siege in affecting both constructs, as well as mental distress among our participants. In fact, higher scores on the scale measuring effect of the siege was associated with loss of hope for the future, lack of expectations, and motivation similarly to what emerged from our findings (Afifi *et al*., 2013). Furthermore, our findings showed how living under conditions of isolation and constant burden undermined sources of resilience that help individuals to adjust to ongoing traumatic realities and maintain good psychological functioning. As previously stressed by other studies carried out in Gaza Strip, the results of our research showed the more the siege affected individuals' living conditions, the lower resulted their resilience and hope, and the greater their mental distress, including anxiety, traumatic stress, and depression (Thabet *et al*., 2009; Thabet and Vostanis, 2011; Thabet *et al*., 2015).

Unlike previous research (Thabet *et al*., 2009), male students had higher hopelessness scores than female students. In fact, the lack of hope among male participants reached cut-off scores for suicide risk, making this dimension a gender specific and relevant factor to be considered in clinical practice and preventive work (Veronese *et al*., 2021). Moreover, males appeared to be more affected by the blockade than females and reported greater psychological distress. The recent rise of unemployment in the region and the increased lack of opportunities may have contributed to undermining cultural roles of male students (Greenwood and Thomson, 2020). As a result of economic and social disruption, men may perceive themselves as under-equipped to meet the cultural pressures for social and familial recognition. They may be unable to find the necessary resources to marry and found a family, both of which are social and cultural expectations (Muhanna, 2016; MacKenzie and Foster, 2017). On the contrary, women in Gaza have positioned themselves as actors in resisting the Israeli occupation, fostering their and their children's psychological well-being, and displaying a higher level of resiliency (Veronese *et al*., 2019b; Veronese *et al*., 2020).

Unfortunately, despite the authorities in Gaza recognize an increased alert on risk of suicide among youths, especially boys, there are no systematic data exhaustively explaining the phenomenon. In the near future, further research will try to explain the factors that can increase the risk of suicide among students in Gaza, moving from our drafted although alarming results. Furthermore, we recommend implementing epidemiological studies on the risk of suicide to help the authority in better understand and act to contrast a relatively new social issue in Gaza.

The effect of the siege not only compromises the mental health of the Gaza population (Bruno *et al*., 2019), but also undermines students' sense of hope (AlDahdouh, 2020) and resilience (Brück *et al*., 2019), thus adding to their psychological burden. Years of blockade have progressively reduced the resources and skills of survival among students in Gaza that can help resist and confront political oppression and military violence (Veronese and Barola, 2018; Kteily-Hawa *et al*., 2020).

The survey was limited to university students, which may have limited generalizability to the Gaza population. The sample size and the cross-sectional nature of our study can be considered additional factors that limit our ability to make claims of causality among the variables. Youths in Gaza represent a large percentage of the general population (21.34%), where the median age is 14.4 years old (Index Mundi, 2019). Accordingly, our sample may represent an interesting glimpse on the Gaza society. Future research should be addressed in understanding the effect of the siege on young people, elderly persons, and middle-aged individuals. Another caveat is that the measures we used in the study were not developed in the local population, which may have limited their ability to assess indigenous constructs both regarding mental distress and resilience (Giacaman, 2019; Hammad and Tribe, 2020). In the future, ethnographic and mixed method studies will allow a better understanding of dimensions and domains of the psychological distress and their association with the ongoing blockade of Gaza.

In terms of ecological validity, the evidence from the study can be used in two ways. First of all, in order to assist in developing guidelines and policies options for contextual-based psycho-social and community psychosocial interventions that are more efficient and that can respond to the local specificities of living under siege (Diab *et al*., 2018). The second point was linked to the opportunity of shedding new light on theoretical and psychological mechanisms that characterized the personal experience of living under the condition of prolonged and long-standing siege.

## Conclusion

Our findings draw attention to how the ongoing violation of human rights influences the mental health of people in Gaza (Diab *et al*., 2018; Diab *et al*., 2020). In our study, the ongoing siege emerged as a crucial risk factor that contributes to undermining the Gaza students' psychological well-being. Loss of hope and disrupted sources of resilience exacerbate the mental distress of the participants to our study. In 2016, The United Nations declared that by 2020 the Gaza Strip would be unlivable (UNDP, 2016). That time has now arrived and the current situation gravely compromises the psychological functioning and capabilities of adjustment among youth. Accordingly, interventions aimed at promoting mental health in Gaza cannot avoid including a perspective that includes human rights and social justice (Kteily-Hawa *et al*., 2020). The main lesson learnt from our study is that people deprived of their freedom and besieged for long periods in an environmentally, politically, and socially compromised condition, are at risk to lose fundamental protective factors and at risk of being compromised in their mental well-being. Ultimately, our study might challenge the mainstream notion that deteriorated mental health conditions will compromise individuals' quality of life (Gerino *et al*., 2017). On the contrary, in the case of the Gaza Strip and other places affected by chronic violations of basic human rights, undermined living conditions, and quality of life will contribute to weakening source of resilience, hope, and might increase risk of psychological suffering (Veronese *et al*., 2019a).

Thus, the traditional instruments that clinical and psycho-social interventions have used to intervene in Gaza and other zones affected by chronic warfare risk to be ineffective if the environmental conditions did not change. This study invites mental health providers to fully consider the political antecedent and determinants of the human suffering, to implement interventions in coordination with human rights defenders, political actors, and influencers capable of advocating for and promoting social suffering in the context of systematic and structural violation of human rights.

Specifically, in the case of Gaza, similar to other human rights activists, mental health providers' should advocate for an immediate lift of the blockade and the restoration of international law in the region to protect civilians' dignity, mental well-being, and human security.
